# Supplementary material for: Whole-Exome Screening and Analysis of Signaling Pathways in Multiple Endocrine Neoplasia Type 1 Patients with Different Outcomes: Insights into Cellular Mechanisms and Possible Functional Implications
Source: Int J Mol Sci. 2024 Jan 15;25(2):1065. doi: 10.3390/ijms25021065 (PMC10816043; doi:10.3390/ijms25021065)
Supplement: Supplementary file 1 [file ijms-25-01065-s001.zip › ijms-2784639-supplementary.pdf]

Supplementary Table S1. Pathways shared between all patient groups in the MEN1 pituitary analysis. P-values for the pathway in each group are stated. Genes with non-neutral variants for each patient group are listed. Genes that repeatedly contributed to the identification of pathways are mentioned on a shaded background. In brackets, the frequencies of variant alleles [GnomAD, non-Finnish European population] identified in the genes are presented.

Pathway - identifier	Family A	Patient X5	Patient X15
Alzheimer's disease - WP5124			
	P=0.0335	P=0.0671	P=0.1524
	KIF5C (0.0018)	*APOE* (0.0767)	*CACNA1F* (0.0247)
	LRP1 (unknown)	*ATP2A3* (0.0020)	*CACNA1S* (0.1294)
		LRP5 (0.0522)	
		CACNA1S (0.1294, 3.52E-5, 0.0502)	
Brain-Derived Neurotrophic Factor (BDNF) signaling pathway - WP2380			
	P=0.1453	P=0.2016	P=0.5212
	NGF (5.74E-4)	*ACACB* (0.0631)	*DLG1* (0.0514)
Burn wound healing - WP5055			
	P=1.97E-4	P=0.0219	P=0.0159
	COL1A1 (5.06E-3)	*FN1* (4.34E-3)	*FN1* (3.10E-5)
	MMP1 (3.88E-5)		*TNC* (2.56E-3)
Ciliary landscape - WP4352			
	P=0.2057	P=0.3434	P=0.0232
	USH1C (0.000*)	*CDH23* (6.41E-4)	*EXOC2* (0.1016)
			LZTFL1 (0.0830)
	*detected only in the Latino/Admixed American population		
Development of ureteric collection system - WP5053			
	P=0.0622	P=2.36E-5	P=1.31E-5
	FREM2 (0.0499)	*FRAS1* (0.0201)	*BMPR1A* (8.79E-6)
		FREM1 (0.2456)	*FREM1* (0.2456)
		GPC3 (unknown)	*GLI3* (0.0502)
		PBX1 (0.2192)	*PBX1* (0.2192)
DNA IR-damage and cellular response via ATR - WP4016			
	P=0.0845	P=0.3740	P=0.0644
	FANCI (1.08E-3)	*FANCA* (3.88E-3)	*FANCI* (0.0833)
			HERC2 (5.06E-3)
DNA repair pathways, full network - WP4946			
	P=0.1207	P=0.0311	P=0.1217
	FANCI (1.08E-3)	*FANCA* (3.88E-3)	*FANCI* (0.0833)
		PARP2 (0.0247)	*POLL* (0.2233)
		POLE (0.0144)	
Focal Adhesion - WP306			

P=0.1934	P=2.06E-5	P=8.46E-6
COL1A1 (5.06E-3)	*FLNB* (0.2467, 0.2472)	*COL4A4* (0.4798)
	FN1 (4.34E-3)	*FN1* (3.10E-5)
	LAMC2 (0.0868)	*LAMA1* (6.25E-3)
	MET (0.0118)	*LAMC2* (0.0868)
	THBS1 (8.28E-4)	*MET* (0.0118)
	TLN2 (4.12E-3)	*RELN* (0.0160)
		TNC (2.56E-3)
Focal Adhesion-PI3K-Akt-mTOR-signaling pathway - WP3932		
P=0.0420	P=3.70E-4	P=4.53E-7
COL1A1 (5.06E-3)	*FN1* (4.34E-3)	*COL4A4* (0.4798)
NGF (5.74E-4)	*LAMC2* (0.0868)	*FN1* (3.10E-5)
	MET (0.0118)	*HIF1A* (0.1049)
	TBC1D1 (0.0133)	*LAMA1* (6.25E-3)
	THBS1 (8.28E-4)	*LAMC2* (0.0868)
		MET (0.0118)
		RELN (0.0160)
		TNC (2.56E-3)
Hepatitis C and Hepatocellular Carcinoma - WP3646		
P=0.0530	P=0.2510	P=0.2254
MMP1 (3.88E-5)	*CXCR1* (7.38E-4)	*HIF1A* (0.1049)
Hypothesized Pathways in Pathogenesis of Cardiovascular Disease - WP3668		
P=0.0269	P=0.1345	P=0.1199
LTBP1 (0.0184)	*FBN2* (1.68E-3)	*FBN2* (3.15E-3)
IL-18 signaling pathway - WP4754		
P=0.0033	P=0.0217	P=0.4074
COL1A1 (5.06E-3)	*ACACB* (0.0631)	*FN1* (3.10E-5)
MMP1 (3.88E-5)	*ACADS* (0.0442)	
PTPRZ1 (6.48E-5)	*FN1* (4.34E-3)	
	HADH (8.16E-3)	
Inflammatory Response Pathway - WP453		
P=0.0332	P=2.99E-5	P=5.27E-4
COL1A1 (5.06E-3)	*FN1* (4.34E-3)	*FN1* (3.10E-5)
	LAMC2 (0.0868)	*LAMC2* (0.0868)
	THBS1 (8.28E-4)	
Malignant_pleural_mesothelioma - WP5087		
P=0.0765	P=0.0032	P=1.94E-6
NGF (5.74E-4)	*COL4A3* (0.4822)	*CDH20* (0.1372)
SOST (1.40E-4)	*EZH2* (0.0798)	*COL4A3* (0.4822)
	FN1 (4.34E-3)	*COL4A4* (0.4798)
	LAMC2 (0.0868)	*FN1* (3.10E-5)
	LRP5 (0.0522)	*HIF1A* (0.1049)

	MET (0.0118)	*LAMA1* (6.25E-3)
	SELE (0.1051)	*LAMC2* (0.0868)
		MET (0.0118)
		TERT (0.0301)
MAPK Signaling Pathway - WP382		
P=0.2364	P=0.1712	P=0.3587
NGF (5.74E-4)	*CACNA1S* (0.1294, 3.52E-5, 0.0502)	*CACNA1F* (0.0247)
	FLNB (0.2467, 0.2472)	*CACNA1S* (0.1294)
	HSPA1L (0.000*)	
*detected only in the Asian population		
Metapathway biotransformation Phase I and II - WP702		
P=0.1836	P=1.36E-5	P=0.0154
CHST12 (8.94E-6)	*CHST5* (0.2044)	*AKR7A2* (0.0915)
	CYP27A1 (0.0224)	*EPHX2* (0.0991)
	CYP2C18 (0.1461)	*FMO2* (0.0869)
	CYP4F2 (0.2866)	*GSTO2* (3.10E-4)
	EPHX2 (0.0991)	
	FMO2 (0.0869)	
	FMO5 (0.0109)	
	MGST1 (1.64E-4)	
miR-509-3p alteration of YAP1/ECM axis - WP3967		
P=0.0194	P=0.0988	P=0.0878
COL1A1 (5.06E-3)	*FN1* (4.34E-3)	*FN1* (3.10E-5)
Nanoparticle-mediated activation of receptor signaling - WP2643		
P=0.0300	P=0.1494	P=0.1333
COL1A1 (5.06E-3)	*FN1* (4.34E-3)	*FN1* (3.10E-5)
Nephrotic syndrome - WP4758		
P=0.0489	P=0.0290	P=0.0231
ARHGAP24 (2.64E-5)	*COL4A3* (0.4822)	*COL4A3* (0.4822)
	CUBN (0.0384)	*COL4A4* (0.4798)
Neural Crest Differentiation - WP2064		
P=0.1032	P=0.4392	P=0.0925
GFAP (8.34E-3)	*GFAP* (0.0296)	*NOTCH3* (0.0168)
Neuroinflammation and glutamatergic signaling - WP5083		
P=0.0102	P=0.1932	P=0.0351
GFAP (8.34E-3)	*DLAT* (0.3569)	*SHMT1* (1.20E-3)
NGF (5.74E-4)	*GFAP* (0.0296)	
Pathways in clear cell renal cell carcinoma - WP4018		
P=0.0875	P=0.0847	P=9.17E-4
GPI (0.0215)	*ACACB* (0.0631)	*HIF1A* (0.1049)

	ZEB1 (3.32E-3)	*PGM1* (0.1898)
		SHMT1 (1.20E-3)
PI3K-Akt Signaling Pathway - WP4172		
P=0.0518	P=1.59E-4	P=2.23E-8
COL1A1 (5.06E-3)	*COL4A3* (0.4822)	*COL4A3* (0.4822)
NGF (5.74E-4)	*COL6A3* (0.0883)	*COL4A4* (0.4798)
	FN1 (4.34E-3)	*COL6A3* (0.0883)
	LAMC2 (0.0868)	*COL9A2* (3.87E-3)
	MET (0.0118)	*FN1* (3.10E-5)
	PCK1 (0.1216)	*G6PC2* (8.59E-3)
	THBS1 (8.28E-4)	*LAMA1* (6.25E-3)
		LAMC2 (0.0868)
		MET (0.0118)
		RELN (0.0160)
		TNC (2.56E-3)
PPAR signaling pathway - WP3942		
P=0.0714	P=0.0072	P=0.2935
MMP1 (3.88E-5)	*APOA5* (0.0637)	*ACADL* (0.0307)
	CYP27A1 (0.0224)	
	PCK1 (0.1216)	
Prader-Willi and Angelman syndrome - WP3998		
P=0.0704	P=0.0572	P=0.0049
NGF (5.74E-4)	*CDC6* (0.0604)	*HERC2* (5.06E-3)
	KISS1 (0.0379)	*OCA2* (0.0650)
Senescence and Autophagy in Cancer - WP615		
P=0.1101	P=0.1268	P=0.4214
COL1A1 (5.06E-3)	*FN1* (4.34E-3)	*FN1* (3.10E-5)
	THBS1 (8.28E-4)	
Statin inhibition of cholesterol production - WP430		
P=0.0311	P=6.17E-4	P=0.1377
LRP1 (unknown)	*APOA5* (0.0637)	*APOB* (0.2156)
	APOE (0.0767)	
	LCAT (0.0284)	
Sudden Infant Death Syndrome (SIDS) Susceptibility Pathways - WP706		
P=0.1656	P=0.2481	P=0.2074
NGF (5.74E-4)	*CHAT* (0.0650)	*HIF1A* (0.1049)
	PBX1 (0.2192)	*PBX1* (0.2192)
TGF-beta Signaling Pathway - WP366		
P=0.1340	P=0.0073	P=0.0048
MMP1 (3.88E-5)	*FN1* (4.34E-3)	*FN1* (3.10E-5)
	MET (0.0118)	*MET* (0.0118)
	THBS1 (8.28E-4)	*TERT* (0.0301)
	ZEB1 (3.32E-3)	*TNC* (2.56E-3)

VEGFA-VEGFR2 signaling pathway - WP3888						
P=0.3781		P=0.1072		P=0.1814	
PTPRZ1	(6.48E-5)	*ACACB*	(0.0631)	*FN1*	(3.10E-5)
		FLNB	(0.2467, 0.2472)	*TKT*	(7.45E-3)
		FN1	(4.34E-3)	*TRAF3IP2*	(1.22E-3)
		SELE	(0.1051)		

Supplementary Table S2. Pathways shared between all patient groups in the MEN1 adrenal analysis. P-values for the pathway in each group are stated. Genes with non-neutral variants for each patient group are listed. Genes that repeatedly contributed to the identification of pathways are mentioned on a shaded background. In brackets, the frequencies of variant alleles [GnomAD, non-Finnish European population] identified in the genes are presented.

Pathway – identifier			
Family A	Family C	Patient X11	Patient X15
**Selenium micronutrient network - WP15**			
P= 0.1513	P= 0.1657	P= 0.3389	P= 0.3188
APOB (0.0350)	*SELP* (0.000*)	*TXNRD2* (0.3430)	*TXNRD2* (0.3430)
	TXNRD2 (0.3430)		
* identified only in the African/African American population			
**MicroRNAs in cardiomyocyte hypertrophy - WP1544**			
P= 0.1594	P= 0.1744	P= 0.0097	P= 0.3339
NRG1 (2.32E-5)	*NRG1* (0.0441)	*FZD1* (1.30E-4)	*NRG1* (0.0208)
**Neural crest differentiation - WP2064**			
P= 0.0157	P= 0.1899	P= 0.3821	P= 0.0735
MPZ (0.0316)	*COL11A2* (1.09E-4)	*TCF7L1* (0.0595)	*NOTCH3* (0.0168)
NOTCH3 (6.62E-5)			
**Arrhythmogenic right ventricular cardiomyopathy - WP2118**			
P= 0.0088	P= 0.1443	P=4.54E-4	P= 0.0045
CACNB2 (0.1800)	*DSP* (1.77E-3)	*ACTN2* (8.21E-4)	*ACTN2* (3.26E-4)
ITGB4 (2.25E-4)		*CACNA1S* (8.79E-6)	*CACNA1F* (0.0247)
		SGCG (0.1305)	*SGCG* (0.1305)
		TCF7L1 (0.0595)	
**Primary focal segmental glomerulosclerosis FSGS - WP2572**			
P= 0.0084	P= 0.1407	P= 0.0051	P= 0.0411
DNM1 (4.86E-3)	*COL4A4* (0.0367)	*CR1* (0.0282)	*AGRN* (2.05E-3)
ITGB4 (2.25E-4)		*JAG1* (0.0673)	*COL4A4* (0.4798)
		LAMA5 (0.0186, 0.0904)	
**Cardiac Hypertrophic Response - WP2795**			
P= 0.0978	P= 0.1075	P= 0.2288	P= 0.2142
NRG1 (2.32E-5)	*NRG1* (0.0441)	*CDK7* (0.0245)	*NRG1* (0.0208)
**Ectoderm differentiation - WP2858**			
P= 0.0290	P= 0.2539	P= 0.4880	P= 0.0035
SIX6 (4.88E-3)	*PTPRB* *(unknown)*	*TCF7L1* (0.0595)	*BMPR1A* (8.79E-6)
			EDA2R (8.99E-3)
			GLI3 (0.0502)
			ZFHX4 (7.37E-3)
**Apoptosis-related network due to altered Notch3 in ovarian cancer - WP2864**			
P= 0.0961	P= 0.1056	P= 0.0021	P= 0.0234

NRG1	(2.32E-5)	*NRG1*	(0.0441)	*ABL1*	(7.61E-3)	*NRG1*	(0.0208)
				NQO1	(0.1886)		
				TNFRSF10B	(0.0706)		
Vitamin D Receptor Pathway - WP2877							
P= 0.2963		P= 0.3216		P= 0.0592		P= 0.1977	
STEAP4	(0.1942)	*KNG1*	(0.1109)	*KL*	(0.1526)	*HIF1A*	(0.1049)
				NOX1	(0.0272)	*KNG1*	(0.1109)
				STEAP4	(0.1942)		
NRF2 pathway - WP2884							
P= 0.0294		P= 0.0352		P= 0.0047		P= 0.4650	
EPHA3	(1.87E-3)	*NRG1*	(0.0441)	*NQO1*	0.1886)	*NRG1*	(0.0208)
NRG1	(2.32E-5)	*SLC5A2*	(1.55E-5)	*SLC2A6*	(0.0872)		
				SLC39A13	(3.65E-3)		
				SLC39A4	(1.50E-3)		
miRNA targets in ECM and membrane receptors - WP2911							
P= 0.0592		P= 0.0021		P= 0.1427		P= 0.1331	
LAMA4	(0.0645)	*COL5A1*	(1.04E-3)	*COL5A1*	(0.0468)	*FN1*	(3.10E-5)
		COL6A2	(3.15E-5)				
Focal adhesion - WP306							
P= 5.59E-4		P= 0.0084		P= 0.0153		P= 2.66E-4	
ITGB4	(2.25E-4)	*COL4A4*	(0.0367)	*FLNA*	(3.97E-3)	*COL4A4*	(0.4798)
LAMA4	(0.0645)	*COL6A2*	(3.15E-5)	*FLNC*	(9.68E-3)	*FN1*	(3.10E-5)
THBS4	(0.2203)	*TNC*	(7.08E-3)	*LAMA5*	(0.0186, 0.0904)	*LAMA1*	(0.0101)
				RELN	(0.0158)	*RELN*	(0.0160)
						TNC	(2.56E-3)
TGF-beta signaling pathway - WP366							
P= 0.2227		P= 0.0316		P= 0.4704		P= 0.0029	
ITGB4	(2.25E-4)	*TGIF1*	(0.0568)	*TRAP1*	(0.1837)	*FN1*	(3.10E-5)
		TNC	(7.08E-3)			*TERT*	(0.0301)
						TNC	(2.56E-3)
						TRAP1	(0.1837)
Striated muscle contraction - WP383							
P= 0.0699		P= 0.0769		P= 0.0144		P= 6.52E-4	
NEB	(3.59E-4)	*MYBPC1*	(0.1563)	*ACTN2*	(8.21E-4)	*ACTN2*	(3.26E-4)
				TTN	(4.96E-5)	*NEB*	(0.3281)
						TTN	(0.3075)
MYD88 distinct input-output pathway - WP3877							
P= 0.0337		P= 0.0372		P= 0.0829		P= 0.0772	
TLR10	(0.3691)	*TLR10*	(0.3691)	*TLR10*	(0.3691)	*TLR10*	(0.3691)
VEGFA-VEGFR2 signaling pathway - WP3888							
P= 0.2002		P= 0.2317		P= 0.0052		P= 0.3046	

ACACB	(0.1943)	*TRAF3IP2*	(0.0740)	*ABL1*	(7.61E-3)	*FN1*	(3.10E-5)
EPB41	(0.0492, 7.66E-3)			*BIN1*	(0.0156)	*TKT*	(7.45E-3)
				JAG1	(0.0673)	*TRAF3IP2*	(1.22E-3)
				KL	(0.1526)		
				NCF2	(0.0487)		
				SCUBE2	(0.0777)		
				TKT	(1.19E-3)		
Amino acid metabolism - WP3925							
P= 0.1594		P= 0.1744		P= 0.3547		P= 0.0623	
HDC	(0.0273)	*IDH1*	(0.0136)	*FAH*	(0.0234)	*G6PC2*	(8.59E-3)
						HDC	(0.0273)
Focal adhesion-PI3K-Akt-mTOR-signaling pathway - WP3932							
P= 2.37E-5		P= 4.32E-4		P= 0.0150		P= 6.32E-5	
HSP90B1	(0.0176)	*COL11A2*	(1.09E-4)	*COL5A1*	(0.0468)	*COL4A4*	(0.4798)
ITGAE	(0.0533)	*COL4A4*	(0.0367)	*LAMA5*	(0.0186, 0.0904)	*FN1*	(3.10E-5)
ITGB4	(2.25E-4)	*COL5A1*	(1.04E-3)	*NGF*	(0.0172)	*HIF1A*	(0.1049)
LAMA4	(0.0645)	*COL6A2*	(3.15E-5)	*RELN*	(0.0158)	*LAMA1*	(0.0101)
THBS4	(0.2203)	*TNC*	(7.08E-3)			*RELN*	(0.0160)
						TNC	(2.56E-3)
TYROBP Causal Network - WP3945							
P= 0.1081		P= 0.0071		P= 0.2507		P= 0.2349	
TCIRG1	(0.0616)	*TCIRG1*	(1.71E-4)	*NCF2*	(0.0487)	*DPYD*	(0.1018)
PI3K-Akt signaling pathway - WP4172							
P= 4.65E-4		P= 0.0056		P= 0.0235		P= 2.18E-5	
HSP90B1	(0.0176)	*COL4A4*	(0.0367)	*LAMA5*	(0.0186, 0.0904)	*COL4A4*	(0.4798)
ITGB4	(2.25E-4)	*COL6A2*	(3.15E-5)	*NGF*	(0.0172)	*COL9A2*	(3.87E-3)
LAMA4	(0.0645)	*TNC*	(7.08E-3)	*RELN*	(0.0158)	*FN1*	(3.10E-5)
THBS4	(0.2203)					*G6PC2*	(8.59E-3)
						LAMA1	(0.0101)
						RELN	(0.0160)
						TNC	(2.56E-3)
EMT in colorectal cancer - WP4239							
P= 0.2632		P= 0.0448		P= 0.1792		P= 7.76E-4	
NOTCH3	(6.62E-5)	*COL4A4*	(0.0367)	*FZD1*	(1.30E-4)	*CLDN20*	(0.0154)
		DSP	(1.77E-3)	*JAG1*	(0.0673)	*COL4A4*	(0.4798)
						FN1	(3.10E-5)
						HIF1A	(0.1049)
						NOTCH3	(0.0168)
Ciliary landscape - WP4352							
P= 0.3318		P= 0.3592		P= 0.0036		P= 0.2417	
CDH23	(0.1719)	*BBS5*	(1.49E-3)	*APC*	(5.45E-5)	*CDH23*	(0.2679)

		BBS5 (8.68E-3)	*EXOC2* (0.1016)
		CDH23 (0.0279, 0.2679)	
		IFT122 (6.37E-3)	

GPCRs, Class A Rhodopsin-like - WP455

P= 0.3928	P= 0.1046	P= 0.1310	P= 0.1113
MTNR1A (0.0145)	*AVPR1A* (0.0126)	*ADRA1A* (0.0105)	*HTR1B* (0.0136)
		MC1R (0.0718)	*MC1R* (0.0797)
			MTNR1B (1.44E-3)

Small cell lung cancer - WP4658

P= 0.1658	P= 0.1813	P= 0.3670	P= 8.81E-4
LAMA4 (0.0645)	*COL4A4* (0.0367)	*LAMA5* (0.0186, 0.0904)	*COL4A4* (0.4798)
			FN1 (3.10E-5)
			LAMA1 (0.0101)

Male infertility - WP4673

P= 0.2374	P= 0.2586	P= 0.0312	P= 0.1320
APOB (0.0350)	*PEMT* (1.05E-3)	*AHRR* (0.3994)	*AHRR* (0.3994)
		AR (2.48E-3)	*SHMT1* (1.20E-3)
		NQO1 0.1886)	

IL-18 signaling pathway - WP4754

P= 0.0962	P= 0.4392	P= 0.1452	P= 0.7066
ACACB (0.1943)	*TRPM7* (0.000*)	*BIN1* (0.0156)	*FN1* (3.10E-5)
EPB41 (0.0492, 7.66E-3)		*NCF2* (0.0487)	
		NOX1 (0.0272)	

* identified only in the African/African American population

Proximal tubule transport - WP4917

P= 0.1013	P= 0.1112	P= 0.2362	P= 0.2211
ATP6V1B1 (0.0232)	*SLC5A2* (1.55E-5)	*ATP6V0A4* (7.12E-3, 0.0457)	*SLC7A9* (0.3356)

Malignant pleural mesothelioma - WP5087

P= 0.1906	P= 0.5886	P= 2.12E-4	P= 0.0029
ITGB4 (2.25E-4)	*COL4A4* (0.0367)	*APC* (5.45E-5)	*COL4A4* (0.4798)
LAMA4 (0.0645)		*CDK7* (0.0245)	*FN1* (3.10E-5)
		FZD1 (1.30E-4)	*HIF1A* (0.1049)
		LAMA5 (0.0186, 0.0904)	*LAMA1* (0.0101)
		NGF (0.0172)	*TERT* (0.0301)
		TCF7L1 (0.0595)	

ErbB Signaling Pathway - WP673

P= 0.1578	P= 0.0155	P= 0.3516	P= 0.3309
NRG1 (2.32E-5)	*ERBB4* *(unknown)*	*ABL1* (7.61E-3)	*NRG1* (0.0208)
	NRG1 (0.0441)		

Sudden Infant Death Syndrome (SIDS) susceptibility pathways - WP706							
P= 0.2716		P= 0.0479		P= 0.0013		P= 0.5238	
HSP90B1	(0.0176)	*NR3C1*	(0.0266)	*AR*	(2.48E-3)	*HIF1A*	(0.1049)
				MBD1	(0.2049)		
				NGF	(0.0172)		
				RET	(9.06E-4)		
				TPH2	(1.48E-3)		
